# A single circular chromosome yeast

**DOI:** 10.1038/s41422-018-0110-y

**Published:** 2018-12-17

**Authors:** Yangyang Shao, Ning Lu, Chen Cai, Fan Zhou, Shanshan Wang, Zhihu Zhao, Guoping Zhao, Jin-Qiu Zhou, Xiaoli Xue, Zhongjun Qin

**Affiliations:** 1 Key Laboratory of Synthetic Biology, CAS Center for Excellence in Molecular Plant Sciences, Shanghai Institute of Plant Physiology and Ecology, Chinese Academy of Sciences, 200032 Shanghai, China; 2 University of Chinese Academy of Sciences, 100049 Beijing, China; 3 The State Key Laboratory of Molecular Biology, CAS Center for Excellence in Molecular Cell Science, Shanghai Institute of Biochemistry and Cell Biology, Chinese Academy of Sciences, 200031 Shanghai, China; 4 School of Life Science and Technology, ShanghaiTech University, 201210 Shanghai, China; 5 Wuhan Frasergen Bioinformatics Co, 430075 Wuhan, China; 6 Beijing Institute of Biotechnology, 100071 Beijing, China; 7 Shanghai-MOST Key Laboratory of Health and Disease Genomics, Chinese National Human Genome Center at Shanghai, 201203 Shanghai, China; 8 Department of Microbiology and Li Ka Shing Institute of Health Sciences, The Chinese University of Hong Kong, Prince of Wales Hospital, Shatin, New Territories, Hong Kong SAR, China; 9 State Key Laboratory of Genetic Engineering, Department of Microbiology, School of Life Sciences and Institute of Biomedical Sciences, Fudan University, 200032 Shanghai, China

**Keywords:** Chromosomes, Chromosomes

Dear editor,

Most of the prokaryotic cells contain a single circular chromosome. In contrast, the eukaryotic cells usually contain multiple linear chromosomes. Recently, we artificially created a single linear chromosome yeast strain SY14 from native 16 chromosomes in a haploid *Saccharomyces cerevisiae*, which displays minor fitness defects.^1^ In this study, we have created a new yeast strain which contains a single circular chromosome and apparently has not been found in nature.

We used a CRISPR-Cas9 method to induce double-stranded DNA breaks (DSBs) at the regions proximal to two telomeres of the linear chromosome of SY14 (Fig. 1a). Through endogenous homologous recombination, the two DSBs ends were ligated with a donor DNA fragment (Fig. 1a) and this resulted in a new strain designated SY15, which contained a single circular chromosome (Fig. 1a). Immuno-staining of myc-tagged telomere binding protein Sir2^2^ showed that one or two telomere signals seen in the SY14 cells were not detected in the SY15 cells (Fig. 1b), suggesting no telomere in SY15. A pulsed-field gel electrophoresis (PFGE) analysis revealed a 1193 kb band in SY15 which was resulted from the fusion of Chr. X and XVI (Supplementary Information, Fig. S1). Both SY14 and SY15 cells showed no detectable changes in the restriction enzyme digestion pattern of their genomes compared with their descendant cells at passage 100, suggesting that the single chromosome yeasts are able to maintain stable genomes.

**Fig. 1:**
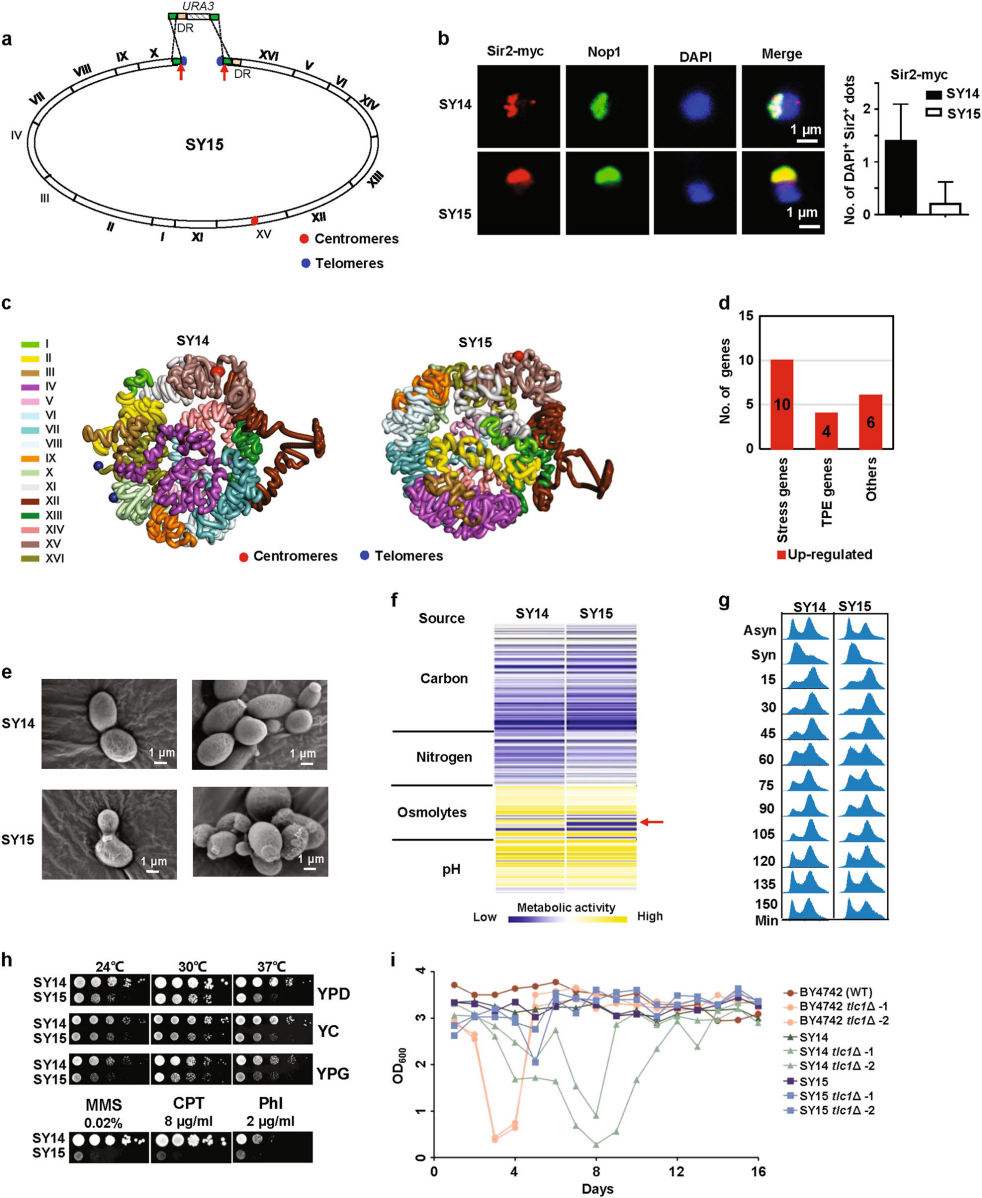
Characterization of the single circular chromosome yeast SY15. **a** Construction of the SY15 strain. Ligation of two chromosome ends via both CRISPR-Cas9 induced DSBs and homologous recombination. The red arrowheads indicated the cutting sites of Cas9. DR: direct repeat. The *URA3* selection marker was further deleted via homologous recombination of two DR regions by negative selection. **b** The myc-tagged telomere binding protein Sir2 was detected with polyclonal anti-myc antibody and Cy3-conjugated (red) secondary antibody. Nop1, a nucleolar protein, was detected with a monoclonal anti-Nop1 antibody and Alexa 488-conjugated (green) secondary antibody. DNA was stained by DAPI (blue).^1^ **c** 3D conformation of the SY15 genome in comparison to that of SY14. **d** Classification of differentially expressed genes, defined as those with log2 (fold change) ≥1 and *P*<0.05 in SY15 compared to SY14. Data were collected from three biological replicates. **e** Scanning electron microscopy pictures of SY14 and SY15 cells. Representative images from three independent experiments. **f** Heatmap of the Phenotype Microarray profiles of SY14 and SY15 cells. Low to high metabolic activities are depicted by a color spectrum from light blue to yellow. Data were collected from two biological replicates. **g** Cell cycle analysis. The yeast cells were synchronized with hydroxyurea and the progression of the cell cycle was analyzed by flow cytometry. Data are representative of two independent experiments. **h** Fitness analysis of SY15 cells under various growth conditions. Representative results of three independent experiments. **i** Senescence assay in liquid medium. The growth of wild type BY4742 (dark brown), SY14 (dark green), SY15 (dark blue), BY4742 *tlc1*Δ (light brown), SY14 *tlc1*Δ (light green), SY15 *tlc1*Δ (light blue) strains were monitored for 16 days. Every 24 h, the growth of the strains was measured in the value of OD_600_. The diluted cultures were started from OD_600_ = 0.01. For each strain, two clones were examined

The chromosome conformation capture (3 C)-derived Hi-C assay^3^ revealed that the circular chromosome in SY15 displayed similar globular configurations to the linear chromosome in SY14 (Fig. 1c). In SY15, the direct joining of two ends of the single chromosome (Supplementary Information, Fig. S2a) resulted in strong interactions of adjacent regions (Supplementary Information, Fig. S2b). Despite that SY15 lost 46% chromosomal interactions as compared with SY14 and gained 13.5% new interactions (Supplementary Information, Fig. S2c), only 20 genes (0.3% of 5815 genes) were differentially expressed (log_2_ (fold change) ≥1 and Padj<0.05) when gene expression profiles of SY15 and SY14 cells were compared (Fig. 1d, Supplementary Information, Table S1). Specifically, 10 genes involved in stress responses were up-regulated in SY15, suggesting that chromosome circularization might have introduced new stresses for yeast cells. Four genes (YPL277C, YPL278C, FEX2, and HSP32) adjacent to the deleted telomeres were up-regulated in SY15 due to the loss of the telomere position effect (TPE).^4^

SY15 and SY14 cells were similar in both size and shape (Fig. 1e), however, a slightly higher ratio (2.2% vs 0.6% at OD_600_ = 1.0) of abnormal long-shape cells was observed in SY15 (Supplementary Information, Fig. S3a, b). Phenotype microarray (PM) analysis showed that SY15 and SY14 cells had comparable metabolic activities for 190 carbon sources, 95 nitrogen sources and in 96 pH conditions (Fig. 1f). A modest reduction of metabolic activities under osmolytes conditions (Fig. 1f), e.g., under high concentration (8–10%) of sodium chloride (Supplementary Information, Fig. S4) was detected in SY15 compared to SY14. SY15 cells could undergo cell division as SY14 cells (Fig. 1g), however, SY15 cells displayed a modest reduction of growth rate in both solid (Fig. 1h, higher panels) and liquid media (Supplementary Information, Fig. S5a) and were quickly outcompeted by SY14 cells when they were co-cultured (Supplementary Information, Fig. S5b), indicating a reduced fitness of the single circular chromosome yeast. Notably, when treated with genotoxic chemicals, such as methyl methanesulfonate (MMS), camptothecin (CPT), and phleomycin (Phl), SY15 cells could hardly grow (Fig. 1h, lower panels). These results suggest that the circularized chromosome has introduced more hurdles for cell functions. It is known that when subjected to stress some of the yeast chromosomes (i.e. chromosome III) are transiently duplicated in order to increase the expression of genes on these chromosomes.^5^ This of course would not be feasible for the yeast strains carrying a single linear or circular chromosome, which may explain the reduced stress tolerance of the single chromosome yeasts reported in this study and in our previous study.^1^

We further examined whether SY15 cells could undergo reproduction sexually. SY15^α^ cells were still capable of mating with the opposite mating type SY15^a^ cells, and formed diploid cells (SY15^α^/ SY15^a^). However, the mating efficiency of SY15^α^ and SY15^a^ cells was ~10 times lower than that of SY14^α^ and SY14^a^ cells. Moreover, the SY15^α^/SY15^a^ diploid cells were unstable, about 15–44% of SY15^α^/ SY15^a^ diploid cells spontaneously converted to haploid cells under normal cultivation conditions. When SY15^α^/SY15^a^ cells cultured in sporulation medium, no tetrads were detected among ~200 examined cells, suggesting that SY15^α^/SY15^a^ cells have difficulty in meiosis.

Next, we deleted *TLC1* gene, which encodes the RNA template component of telomerase^6^ and is essential for telomere replication, in the wide-type BY4742 (32 telomeres), SY14 (2 telomeres) and SY15 (no telomere) strains. The SY14 *tlc1*Δ cells senesced at the fourth re-streak (~100 generations) on the plate (Supplementary Information, Fig. S6a) and the eighth passage in liquid medium (Fig. 1i), which was delayed compared to BY4742 *tlc1*Δ cells. SY14 *tlc1*Δ survivors were gradually emerged in both solid and liquid culture (Fig. 1i, Supplementary Information, Fig. S6a). In contrast, the SY15 *tlc1*Δ cells did not show a decline of growth in either solid or liquid medium (Fig. 1i, Supplementary Information, Fig. S6a). Telomere Southern hybridization revealed that telomeres of SY14 *tlc1*Δ cells shortened along cell passages, and reached to critical length at day 8 when cells were at the senescent state (Supplementary Information, Fig. S6b), indicating that telomere erosion caused cellular senescence. Interestingly, the hybridization signals detected in SY14 *tlc1*Δ survivors (passages 9 and 11) were quite similar to those of SY15 *tlc1*Δ cells (Supplementary Information, Fig. S6b), suggesting that the eroded chromosome ends of SY14 *tlc1*Δ cells fused together. These results indicated that yeast cells with a single circular chromosome could bypass the telomerase-dependent senescence. It will be intriguing to know whether chromosome circularization affects either replicative or chronological aging of yeast cells.

The SY15 strain displays reduced cell growth rate and fitness at conditions tested in this study. The impaired cell growth was also reported in other yeast strains with circularization of chromosomes.^7–9^ We speculated that the severe reduction of SY15 fitness could be attributed to the difficulties in replicating and/or segregating the circular chromosome. Bacteria with a circular chromosome usually replicates its genome from a single replication origin.^10^ But most archaea with circular chromosomes replicate their DNA using multiple origins,^11^ although the controlling mechanism is not well understood. We speculate that the yeast with a single circular chromosome may also replicate its genome using multiple origins, but this speculation awaits future investigations.

From the evolution point of view, the linear chromosomes are thought to facilitate an organism to produce its progenies sexually. However, the emerging of telomeres has imposed many difficulties in cell survival, because telomeres have to be protected by specialized protein complex to avoid fusion and degradation of the linear chromosomal ends. Additionally, due to the end replication problem, telomere replication requires specialized enzyme, i.e. telomerase. Therefore, the evolvement of linear chromosome, as well as telomeres and telomerase, for an organism might be a trade-off for gaining more fitness to the environmental challenges.

## Electronic supplementary material

Supplementary Information

## Electronic supplementary material

**Supplementary information** accompanies for this paper at 10.1038/s41422-018-0110-y.
