# Supplementary material for: A single circular chromosome yeast
Source: Cell Res. 2018 Dec 17;29(1):87–9. doi: 10.1038/s41422-018-0110-y (PMC6318310; doi:10.1038/s41422-018-0110-y)
Supplement: Supplementary file 1 — Supplementary Information [file 41422_2018_110_MOESM1_ESM.pdf]

Supplementary information

A single circular chromosome yeast

Yangyang Shao[1,2#], Ning Lu[1,2#], Chen Cai[2,3,9#], Fan Zhou[4#], Shanshan Wang[3], Zhihu Zhao[5*], Guoping Zhao[1,6,7,8*], Jin-Qiu Zhou[3,9*], Xiaoli Xue[1*], Zhongjun Qin[1*]

Supplementary figures and tables

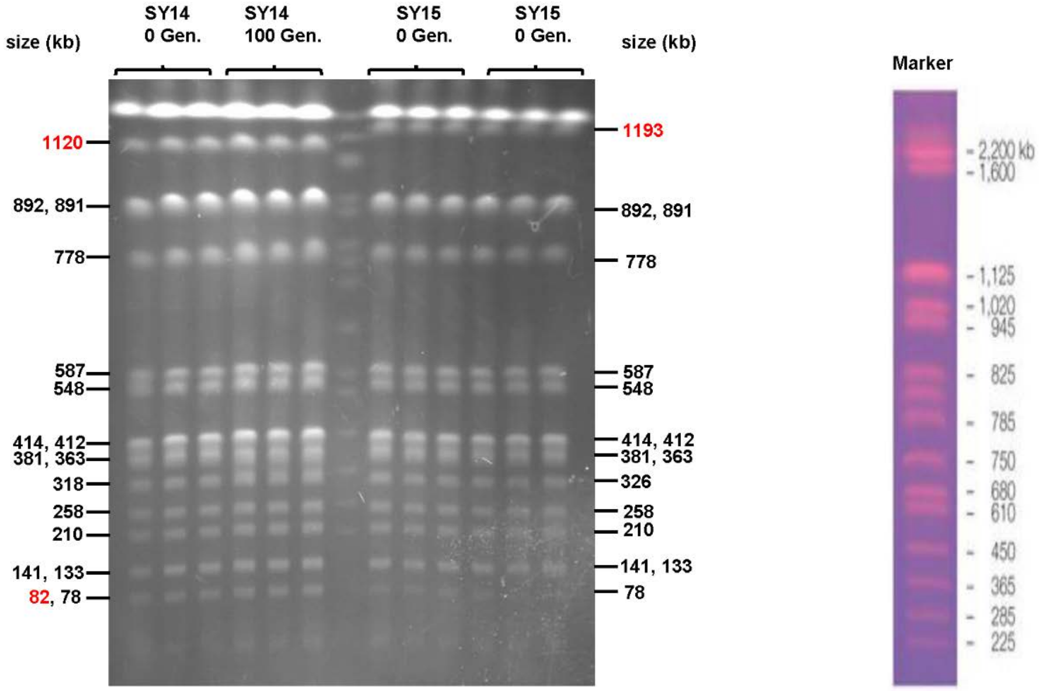

Figure S1 The pulsed-field gel electrophoresis (PFGE) analysis of *Fse*I-digested chromosomal DNA of SY14 and SY15 cells. The genomic DNA of SY14 and SY15

cells were digested by *Fse*I, and separated on a Bio-rad CHEF-DR III system in 1.0% Pulsed Field Certified Agarose gel in 0.5x TBE at 8 °C with a 60 sec switch time in 23 hours and 90 sec switch time in 12.5 hours. The *Fse*I digestion of SY14 genome released DNA fragments with sizes of 1569, 1446, 1050 (+~1500 kb rDNA), 1120, 892, 891, 778, 587, 548, 414, 412, 381, 363, 318, 258, 210, 141, 133, 82, 78, 27, 25.7 and 0.9 kb. The bands smaller than 50 kb or larger than 1.5 Mb could not be clearly separated from the yeast chromosomal fragments under the experimental condition. The genome DNA digestion patterns of SY15 is similar as that of SY14 except for missing of two DNA bands with size of 82 and 1120 kb, and emergence of a new band with size of 1193 kb, which was resulted from the fusion of Chr. X and XVI (a 9-kb fragment was deleted during chromosome end-to-end fusion). The restriction enzyme digestion pattern showed no detectable changes in the genomes of the SY14 or SY15 cells at 0 and 100 generations.

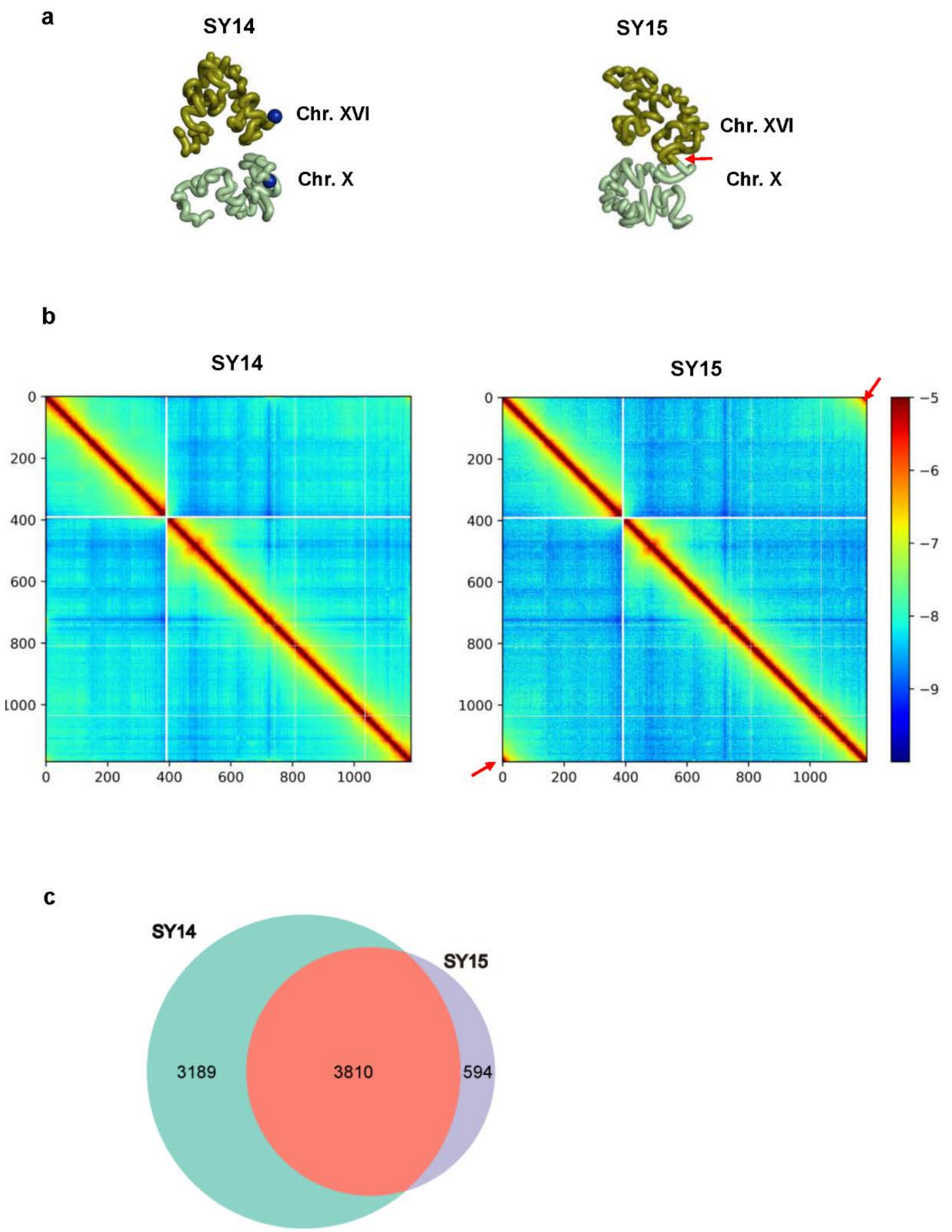

Figure S2 Chromosomal 3D structures and interactions of genomes in SY14 and SY15 strains. **a** Comparison of 3D structures of single chromosomes XVI and X in SY14 and SY15. The red arrowhead at bottom-left and top-right of the heatmap indicated the high interaction frequency near the fusion site of chromosomes XVI and X. **b** The normalized contact heatmaps of SY14 and SY15 genomes with 10-kb

resolution. Low to high interaction frequencies are depicted by a color spectrum from blue to red. **c** Venn diagram for the numbers of significant ($P < 0.01$, $q < 0.01$) chromosomal interactions.

a

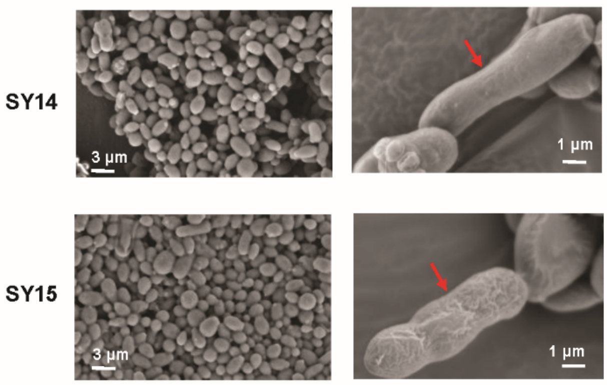

b

OD_{600}=1.0

Cell shape	WT	SY14	SY15
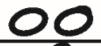	35 (29.7%)	45 (27.9%)	109 (47.2%)
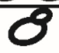	36 (30.5%)	62 (38.5%)	67 (29.0%)
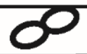	46 (39.0%)	53 (32.9%)	50 (21.6%)
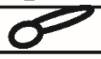	1 (0.8%)	1 (0.6%)	5 (2.2%)
Total	118 (100%)	161 (100%)	231 (100%)

OD_{600}=4.5

Cell shape	SY14MU	SY15MH
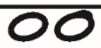	27 (25.7%)	38 (30.6%)
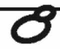	16 (15.2%)	20 (16.1%)
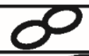	61 (58.1%)	63 (50.8%)
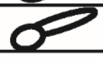	1 (1.0%)	3 (2.4%)
Total	105 (100%)	124 (100%)

Figure S3 Cell morphologic analysis of SY14 and SY15 cells. **a** Scanning electron microscopy pictures of SY14 and SY15 cells. The red arrowheads pointed out the

cells with abnormal long shapes in SY14 and SY15. **b** Quantification of cells with different morphologies. For each strain, the morphologies of 100~200 cells from OD_{600}=1.0 and 4.5 were calculated.

		LN(SY15/SY14)
Carbon	a-D-Glucose	0.04
	D-Fructose	0.07
	D-Mannose	0.08
	D-Xylose	0.03
	D-Galactose	0.07
	D-Ribose	0.01
	Sucrose	0.03
	D-Trehalose	-0.02
	L-Lyxose	-0.14
	D-Arabinose	0.04
	Maltose	-0.01
	D-Cellobiose	0.07
	Lactulose	0.05
	Dulcitol	0.04
	Dextrin	-0.01
	D-Melibiose	0.03
	a-D-Lactose	0.01
	Inosine	0.07
	D-Mannitol	0.08
	Maltotriose	0.04
Nitrogen	Ammonia	-0.26
	L-Asparagine	-0.25
	L-Glutamic Acid	-0.09
	L-Glutamine	-0.03
	L-Arginine	0.07
	L-Aspartic Acid	-0.17
	L-Alanine	-0.05
	L-Serine	0.09
	Urea	-0.10
	L-Tryptophan	-0.09
	Guanosine	0.29
	L-Methionine	0.08
	Gly-Met	0.16
	Met-Ala	0.06
	Ala-Gln	0.00
	Ala-Glu	0.08
	L-Citrulline	0.04
	Ethylenediamine	0.26
	Xanthine	0.01
	Alloxan	0.10
Osmolyte	2% NaCl	-0.09
	4% NaCl	-0.08
	6% NaCl	-0.26
	8% NaCl	-0.43
	10% NaCl	-0.59
pH	pH 3.5	0.11
	pH 4.5	-0.04
	pH 5.5	-0.07
	pH 7	0.15
	pH 8.5	0.08
	pH 10	0.15

>25% decreased

Figure S4 Phenotype microarray (PM) analysis of SY14 and SY15 cells under various conditions. The mean area of growth kinetics of SY15 cells from two

independent experiments was normalized to those of SY14 cells, and the numerical value of its logarithm base 2 is shown. The negative values marked in red color indicated greater than 25% metabolic reduction in SY15 cells.

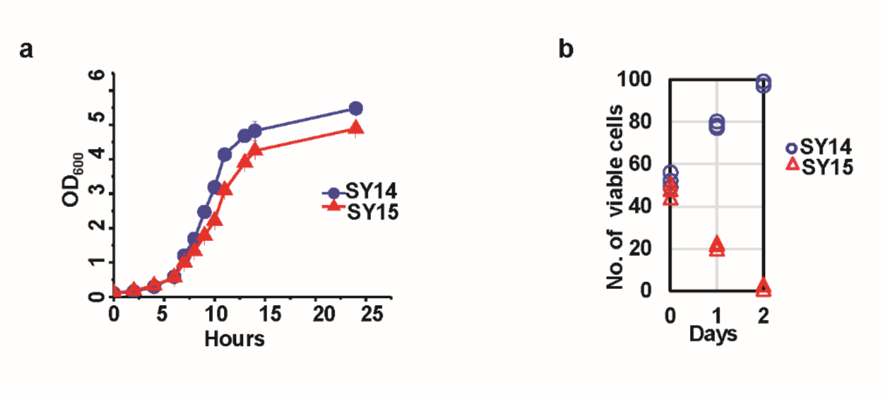

Figure S5 Cell growth and competition of SY14 and SY15. **a** Growth curves of the SY15 (red triangle) and SY14 (blue circle) (mean ± s.e.m.). Three biological replicates were assayed. **b** Growth competition of SY14 and SY15 cells. Data were from three biological replicates.

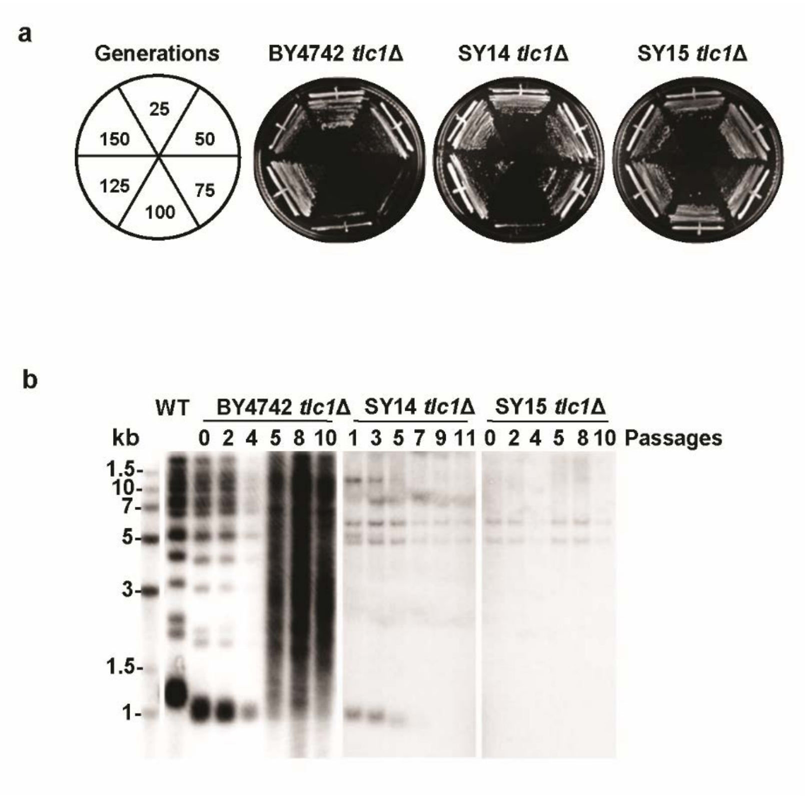

Figure S6 Cellular senescence of SY14 *tlc1Δ* and SY15 *tlc1Δ* strains. **a** Senescence assay of the BY4742 *tlc1Δ*, SY14 *tlc1Δ* and SY15 *tlc1Δ* strains on solid medium. One colony of each strain (labeled on the top) was successively re-streaked on YPD plates for indicated generations until survivors arose. Each streakout counts about 25 generations. **b** Telomere southern hybridization of senescing and survival cells. The genomic DNA of BY4742 *tlc1Δ*, SY14 *tlc1Δ*, and SY15 *tlc1Δ* cells in the liquid cultures (passages indicated) was extracted, digested with *Xho*I and subjected to Southern hybridization with a telomere-specific TG_{1-3} probe.

Table S1 Differentially expressed genes in SY15 compared to SY14 cells.

Gene name	ID	Log2FoldChange (SY15/SY14)	Padj	Gene description
**10 Stress response involving genes**				
STF1	851426	1.00361	0.001926	ATPase-binding protein
GPP2	856791	1.05701	0.000208	Glycerol-1-phosphatase HOR2
HBN1	850331	1.14299	2.26E-05	Putative nitroreductase
SPG4	855134	1.16616	0.008962	Protein required for high temperature survival during stationary phase
SSA4	856840	1.38736	5.79E-06	Hsp70 family chaperone SSA4
RNR3	854744	1.58318	1.72E-08	Ribonucleotide-diphosphate reductase subunit RNR3
HSP12	850532	2.06586	7.51E-13	Lipid-binding protein HSP12
HUG1	854944	2.16781	1.28E-17	Hug1p
HSP26	852364	2.34495	2.50E-11	Chaperone protein HSP26
DDR2	854104	2.36773	4.08E-11	Ddr2p
**4 TPE genes (Chr.XVI-L telomere)**				
YPL277C	855852	1.81537	0.001129	Hypothetical protein
YPL278C	855851	2.00471	3.47E-06	Hypothetical protein
FEX2	855850	3.62663	3.41E-14	Fluoride transporter
HSP32	855849	4.72019	9.01E-09	Glutathione-independent

				methylglyoxalase family protein
**6 Other genes**				
GPM2	851541	1.01485	0.046169	Nonfunctional homolog of Gpm1p phosphoglycerate mutase
STR3	852691	1.02573	0.00172	Peroxisomal cystathionine beta-lyase; converts cystathionine into homocysteine
MFA1	852072	1.03388	0.045395	Mating pheromone a
EGO4	855770	1.14568	7.34E-06	Protein of unknown function; expression is regulated by Msn2p/Msn4p
YCL048W-A	3799968	1.15942	0.028058	Putative protein of unknown function
YLR012C	850699	2.19724	3.26E-05	Putative protein of unknown function

Methods

Plasmid construction. The genomic target sequences S1 (5′-gtggcatccgttcaagcaaa-3′, PAM: tgg) and S2 (5′-tacagaatctcaaaacaagc-3′, PAM: ggg) were manually selected near the XVI left telomere and X right telomere. The gRNA1 and gRNA2 expression cassettes were assembled by fusion PCR of SNR52 promoter, S1/S2 and SUP 3′ flanking sequences. Then the *Eco*RI/*Bam*HI digested gRNA1 and *Bam*HI/*Not*I digested gRNA2 were ligated to an *Eco*RI/*Not*I digested pHIS426[1] to construct the guide RNA expression plasmid pCgRNA.

CRISPR- Cas9 facilitated circularization of linear single chromosome in SY14. In all, 1μg gRNA expression plasmid pCgRNA (with *HIS3* selection marker) and donor DNA (with 400 bp homology arms, *URA3* selection marker and 200 bp direct repeat (DR) sequences for curated selection marker in the second step) were transformed into SY14 cells containing pCas9[1] (constitutively expressing Cas9, *LEU2* selection marker) using a standard lithium acetate transformation method[2]. Cells were plated on synthetic media without uracil, histidine and leucine (SC-Ura-Leu-His). Positive clones verified by PCR were grown in SC-Ura-Leu-His media to saturation and then transferred to SC-Leu media with 2% galactose and 3% raffinose for 16 hours before plating on SC-Leu containing 1mg/ml 5- FOA[3]. The curation of selection markers and pCgRNA of the positive colonies was verified by PCR analysis and sequencing.

Immunofluorescence. Indirect immunofluorescence of Sir2-myc was performed as described previously[1].

Hi-C library construction and sequencing. The cells were crosslinked with 3% formaldehyde for 10 min at room temperature. Formaldehyde was quenched by glycine for 5 min. Liquid nitrogen grinding method was used to break the wall. DNA was digested by *Mbo*I restriction enzyme overnight, the ends of restriction fragments were labeled with biotinylated nucleotides and ligated. The resulting Hi-C libraries were sequenced by Illumina paired-end (PE) platform.

Hi-C data processing. The Hi-C data of SY14 and SY15 strain were iteratively mapped to the SY14 genome and normalized using ICE (version 1f8815d0cc9e)

method[4]. The heatmaps were also plotted by ICE at 10 kb resolution. The significant interactions ($P<0.01$ and $q<0.01$) was called by Fit-Hi-C[5] (v1.0.1) at 5 kb resolution. The correlation of the two biological replicates for each strain was very high (> 0.97) using QuASAR-Rep[6] analysis (from HiFive[7] v1.5.3). The intersect significant interactions of two biological replicates for each strain were used for comparison. Pastis[8] (v0.1) was used to reconstruct the chromosome 3D structure.

RNA-seq analysis. The exponential growth cells from three biological replicates for SY14 and SY15 were collected and prepared for RNA-seq analysis as described previously[1]. The genes with expression fold change more than 2 and $P <0.05$ were identified as differentially expressed genes in SY15/SY14.

Cells morphology analysis. Yeast cells were grown in YPAD medium overnight to saturation and then transferred to fresh YPAD medium to a final OD_{600} of ~1.0. Cells were harvested and prepared for scanning electron microscope (Zeiss) as described previously[9]. Morphologic abnormalities of yeast cells were observed and calculated from three views (100~200 cells) under ordinary optics microscope (Zeiss).

Phenotypic microarray (PM) analysis. Phenotypic microarray analysis of SY14 and SY15 strains was performed as described previously[1].

Cell growth assay. Overnight cultures were diluted to $OD_{600} = 0.1$ in 25 ml liquid YPAD and cultivated at 30 °C. The growth of cells was measured at the indicated time points. Three replicates were assayed.

Growth competition. The *URA3* and *HIS3* genes were introduced into SY14 and SY15 chromosomes, respectively. The growth competition of SY14 (with *URA3*

knock-in) and SY15 (with *HIS3* knock-in) cells were performed as described previously[1].

Cell cycle. Exponential growth cells were synchronized with 200 mM hydroxyurea for 1.5 hours. Cells were washed, fixed in 70% ethanol at 4 °C overnight, and stained by PicoGreen (Invitrogen).Cell cycle analysis was performed using flow cytometry (Beckman) as previously described[1,10].

Sensitivity tests. Temperature, genotoxin and carbon source sensitivity tests were performed as published previously[1]. Single colonies of the tested strains were cultured in YPD medium overnight at 30 °C. The density of cells was adjusted to OD_{600}=0.8, and tenfold serial dilutions were performed. The resulting diluted liquid cultures were spotted onto YPD, YC and YPG plates and were incubated at three temperatures (24 °C, 30 °C and 37 °C). For genotoxin sensitivity assay, the liquids were spotted onto YPD plates with or without the indicated genotoxins. All the plates were photographed after incubation for 3 to 5 days.

Senescence assays. The RNA component of telomerase encoding gene *TLC1*, was deleted in BY4742, SY14 and SY15 haploid cells respectively. The resulting colonies were streaked onto YPD plates. Single colonies of these strains were chosen to perform senescence assays. Re-streak the colonies onto fresh YPD plates and single colonies normally emerge 2 days (~25 population doublings) at 30 °C. The re-streaking procedure was repeated six times every 2 days. In liquid assays, the cell cultures were diluted with fresh YPD medium to a density of OD_{600} = 0.01 every 24 hours and the optical density was measured at 600 nm.

Telomere Southern hybridization. Southern hybridization of *Saccharomyces cerevisiae* telomeres was performed as described previously[11]. Cells were harvested from liquid cultures, and genomic DNA was purified by phenol/chloroform and digested by restriction enzyme *Xho*I. The digested fragments were separated by electrophoresis in 1.0% agarose gel. The fragments were then transferred onto a Hybond-N+ Nylon membrane (GE Healthcare). The telomeric sequences were hybridized with a TG_{1-3} telomere-specific probe. Probe labeling and Chemiluminescence were performed using DIGHigh Prime DNA Labeling and the Detection Starter Kit II (Roche).

Mating and sporulation assays.

The switching of mating-type of the SY15 strain to MATa was performed similarly as described previously[1]. In addition, the *HIS3* and *URA3* selection marker genes were introduced into SY15 and $SY15^a$ strains, respectively. The exponential growth cells of SY15 and $SY15^a$ were mixed thoroughly and shortly centrifuged before incubating at 30 °C for 1.5 h. Appropriate amount of the cell suspension was plated onto SC-His-Ura (synthetic complete medium without histidine and uracil) to obtain $SY15/SY15^a$ diploid cells.

For sporulation, $SY15/SY15^a$ diploid strains were grown to OD_{600} of ~1.0 in 5 ml liquid YPAD at 30 °C. The pellets were washed three times with 1% potassium acetate and incubated in 5 ml 1% potassium acetate at 220 rpm and 25 °C for 1.5 h. Subsequently, cells were harvested and inoculated into 5 ml of sporulation medium[12] (1% potassium acetate, 0.1% yeast extract,0.05% glucose, 0.005% adenosine, 0.005%

uridine, 0.01% tryptophan, 0.01% leucine and 0.01% histidine) at 220 rpm and 28 °C for 3–5 days.

The transcriptome data can be viewed in the NODE (http://www.biosino.org/node) by pasting the accession OER024568,OER024569 into the text search box or through the URL:http://www.biosino.org/node/run/detail/OER024568
http://www.biosino.org/node/run/detail/OER024569
The HiC sequencing data can be viewed in the NODE by pasting the accession OER024570 into the text search box or through the URL: http://www.biosino.org/node/run/detail/OER024570

1 Shao, Y. *et al.* Creating a functional single-chromosome yeast. *Nature*, doi:10.1038/s41586-018-0382-x (2018).

2 Gietz, R. D. & Schiestl, R. H. High-efficiency yeast transformation using the LiAc/SS carrier DNA/PEG method. *Nature protocols* **2**, 31-34, doi:10.1038/nprot.2007.13 (2007).

3 DiCarlo, J. E. *et al.* Genome engineering in *Saccharomyces cerevisiae* using CRISPR-Cas systems. *Nucleic acids research* **41**, 4336-4343, doi:10.1093/nar/gkt135 (2013).

4 Imakaev, M. *et al.* Iterative correction of Hi-C data reveals hallmarks of chromosome organization. *Nature methods* **9**, 999-1003, doi:10.1038/nmeth.2148 (2012).

5 Ay, F., Bailey, T. L. & Noble, W. S. Statistical confidence estimation for Hi-C data

reveals regulatory chromatin contacts. *Genome research* **24**, 999-1011, doi:10.1101/gr.160374.113 (2014).

6 Yardimci, G. *et al.* Measuring the reproducibility and quality of Hi-C data. *bioRxiv*, doi:10.1101/188755 (2017).

7 Sauria, M. E., Phillips-Cremins, J. E., Corces, V. G. & Taylor, J. HiFive: a tool suite for easy and efficient HiC and 5C data analysis. *Genome biology* **16**, 237, doi:10.1186/s13059-015-0806-y (2015).

8 Varoquaux, N., Ay, F., Noble, W. S. & Vert, J. P. A statistical approach for inferring the 3D structure of the genome. *Bioinformatics* **30**, i26-33, doi:10.1093/bioinformatics/btu268 (2014).

9 Bennis, S., Chami, F., Chami, N., Bouchikhi, T. & Remmal, A. Surface alteration of Saccharomyces cerevisiae induced by thymol and eugenol. *Letters in applied microbiology* **38**, 454-458, doi:10.1111/j.1472-765X.2004.01511.x (2004).

10 Manukyan, A., Abraham, L., Dungrawala, H. & Schneider, B. L. Synchronization of yeast. *Methods in molecular biology* **761**, 173-200, doi:10.1007/978-1-61779-182-6_12 (2011).

11 Hu, Y. *et al.* Telomerase-null survivor screening identifies novel telomere recombination regulators. *PLoS genetics* **9**, e1003208, doi:10.1371/journal.pgen.1003208 (2013).

12 Xie, Z. X. *et al.* "Perfect" designer chromosome V and behavior of a ring derivative. *Science* **355**, doi:10.1126/science.aaf4704 (2017).
